# Multiple oncogenic viruses are present in human breast tissues before development of virus associated breast cancer

**DOI:** 10.1186/s13027-017-0165-2

**Published:** 2017-10-16

**Authors:** James S. Lawson, Wendy K. Glenn

**Affiliations:** 1 School of Biotechnology and Biomolecular Sciences, University of New South Wales, Sydney, 2052 Australia; 2 School of BABS, University of NSW, Sydney, NSW 2110 Australia

**Keywords:** Mouse mammary tumor virus, Bovine leukemia virus, Human papilloma virus, Epstein Barr virus, Benign breast, Breast cancer

## Abstract

**Background:**

Multiple oncogenic viruses including, mouse mammary tumor virus, bovine leukemia virus, human papilloma virus, and Epstein Barr virus, have been identified as separate infectious pathogens in human breast cancer. Here we demonstrate that these four viruses may be present in normal and benign breast tissues 1 to 11 years before the development of same virus breast cancer in the same patients.

**Methods:**

We combined the data we developed during investigations of the individual four oncogenic viruses and breast cancer. Patients who had benign breast biopsies 1–11 years prior to developing breast cancer were identified by pathology reports from a large Australian pathology service (Douglas Hanly Moir Pathology). Archival formalin fixed specimens from these patients were collected. The same archival specimens were used for (i) investigations of mouse mammary tumour virus (also known as human mammary tumour virus) conducted at the Icahn School of Medicine at Mount Sinai, New York and at the University of Pisa, Italy, (ii) bovine leukemia virus conducted at the University of California at Berkeley,(iii) human papilloma virus and Epstein Barr virus conducted at the University of New South Wales, Sydney, Australia.

Seventeen normal breast tissues from cosmetic breast surgery conducted on Australian patients were used as controls. These patients were younger than those with benign and later breast cancer.

**Results:**

Standard and in situ polymerase chain reaction (PCR) methods were used to identify the four viruses. The detailed methods are outlined in the separate publications.: mouse mammary tumor virus, human papilloma virus and Epstein Barr virus (Infect Agent Cancer 12:1, 2017, PLoS One 12:e0179367, 2017, Front Oncol 5:277, 2015, PLoS One 7:e48788, 2012).

Epstein Barr virus and human papilloma virus were identified in the same breast cancer cells by in situ PCR.

Mouse mammary tumour virus was identified in 6 (24%) of 25 benign breast specimens and in 9 (36%) of 25 breast cancer specimens which subsequently developed in the same patients.

Bovine leukemia virus was identified in 18 (78%) of 23 benign breast specimens and in 20 (91%) of 22 subsequent breast cancers in the same patients.

High risk human papilloma viruses were identified in 13 (72%) of 17 benign breast specimens and in 13 (76%) of 17 subsequent breast cancers in the same patients.

Epstein Barr virus was not identified in any benign breast specimens but was identified in 3 (25%) of 12 subsequent breast cancers in the same patients.

Mouse mammary tumour virus 3 (18%), bovine leukemia virus 6 (35%), high risk human papilloma virus 3 (18%) and Epstein Barr virus 5 (29%) were identified in 17 normal control breast specimens.

**Conclusions:**

These findings add to the evidence that multiple oncogenic viruses have potential roles in human breast cancer. This is an important observation because evidence of prior infection before the development of disease is a key criterion when assessing causation.

## Background

Over 40 different viruses have been identified by whole genome sequencing in invasive breast cancers [1]. However, only four of these viruses are known to have potential oncogenic influences in breast cancer. These are mouse mammary tumor virus (MMTV), bovine leukemia virus (BLV), human papilloma virus (HPV) and Epstein Barr virus (EBV). Each of these four oncogenic viruses have been studied as separate infectious pathogens in benign breast tissues and subsequent breast cancer in the same patients (2–5). We have previously demonstrated that MMTV, HPV and EBV may be present in the same breast cancer cells [5].These findings have been recently confirmed in a study of MMTV, HPV and EBV in breast cancer among Pakistan women [6]. MMTV, BLV, high risk for cancer HPVs and EBV have an oncogenic capacity in either animals (MMTV and BLV), or humans (HPVs and EBV), [7–10].

### Mouse mammary tumour-like virus (MMTV)

The potential role of MMTV in human breast cancer has been intensively studied for over 50 years. Recent findings have confirmed the likely role of MMTV in human breast cancer. The evidence is as follows: (i) a meta-analysis of 22 studies concluded that the identification of MMTV–like gene sequences in breast cancer tissues, was associated with a 15 fold increase in breast cancer [11], (ii) the near complete proviral structure of MMTV-like virus has been identified in human breast tumours [12], (iii) MMTV proteins indirectly transform human breast epithelial cells [13], (iv) MMTV viral proteins have been identified in human breast cancer [14], (vi) Wnt-1 oncogene expression is significantly higher in MMTV-like positive compared to MMTV-like negative breast cancer specimens, which parallels high Wnt-1 expression in MMTV positive mouse mammary tumours [15], (vii) MMTV can infect human breast epithelial cells and randomly integrate its’ genomic information [16], (viii) there is a progressive increase in the prevalence of MMTV-like viral sequences in normal breast (nil), normal adjacent to breast cancer (19%), breast hyperplasia (27%) and ductal carcinoma in situ (82%) [17], (x) at a microscopic level, the morphology of MMTV-like positive human breast cancer is very similar to the morphology of MMTV positive mouse mammary tumours [15], (xi) MMTV–like viral sequences have been identified in milk from healthy lactating women and increased prevalence in milk from women at high risk for breast cancer [18], (xii) MMTV-like viral sequences have been identified in the saliva of 27% of normal children, 11% of healthy adults and 57% of adults with breast cancer, which is suggestive of a human to human viral transmission [19], (xiii) MMTV-like viral sequences have been identified in breast cancers which developed in a father, mother and daughter of the same family which is suggestive of an infectious condition [20]. In a recent study MMTV-like virus was identified in benign breast tissues before the development 3 to 10 years later of MMTV positive breast cancer in the same patients [2].

Overall, this evidence is compatible with MMTV having similar influences in both human breast cancer and mouse mammary tumours.

### Bovine leukemia virus (BLV)

BLV is a retrovirus which causes leukemia in up to 5% of BLV infected cattle [3]. BLV infected cattle are found world wide and up to 85% of US dairy cattle are infected [3]. Milk of dairy cows frequently contains a leukogenic virus [21]. The evidence for a role of BLV in human breast cancer is as follows: (i) BLV gene sequences and proteins have been identified in human breast tissues in US, Columbian and Australian women [3, 22]. (ii) In a US based study of 114 breast cancer cases and 112 normal controls, the prevalence of BLV was 59% in breast tumours as compared to 29% of controls (*p* = 0.004) [23], (iii) in a Colombia based study of 43 breast cancer cases and 53 non breast cancer controls the prevalence of BLV was 36% in breast tumours and 45% of controls [22], (iv) in a recent Australian study BLV was present in 74% of benign breast tissues 3–10 years before the development of BLV positive breast cancer in the same women [3].

Contrary to the positive identification of BLV in these studies, BLV was not identified by PCR techniques in breast cancers in Chinese patients nor by whole genome sequencing in European patients [24, 25]. The study on Chinese women has been shown to be flawed [3]. The reason for the negative outcome based on whole genome sequencing is not clear. A plausible reason is that whole genome sequencing techniques are not as sensitive as amplification techniques such as PCR [26].

BLV and BLV-infected cells are present in colostrum and milk of most infected cows. This is the most likely means of transmission from cows to humans. There is a striking geographical correlation between the incidence of breast cancer and milk consumption [3]. Women with lactose intolerance and who have a low consumption of milk and dairy products, have a significantly lower risk of breast cancer than other women and other family members [27]. In addition there are significant correlations between the consumption of beef and breast cancer [28].

This evidence is limited, but overall, these data are compatible with a role for BLV in human breast cancer.

### High risk human papilloma virus (HPV)

There is substantial evidence which indicates a potential role for high risk HPVs in breast cancer. This evidence includes (i) meta-analyses of 22 studies conducted in a wide range of countries indicate that infection with high risk HPVs is associated with an increased risk of breast cancer with an overall odds ratio of 5.4, there are marked differences between countries in the prevalence of high risk HPVs in breast cancer [29], (ii) recent studies in the United Kingdom and Spain also indicate an increased risk of breast cancer associated with HPV infection [30, 31], the risk differs according to HPV type, with 5.7 fold higher risk for HPV 16, 3.6 fold increase for HPV 33 and 3.0 fold increase for HPV 18 [29], (iii) HPVs immortalize and transform human mammary epithelial cells [32], (iii) HPV E6 and E7 oncoproteins have been identified in breast cancers, however, there is a low level of transcription of these oncoproteins which suggests that any influences of HPVs in breast cancer are likely to be indirect [4, 33], (iv) high risk HPVs have been identified in the SK-BR-3 breast cancer cell line [34], (v) HPV type 16 E6 and E7 oncoproteins convert non-invasive and non-metastatic breast cancer cells to invasive and metastatic phenotypes [35], (vi) HPV-positive breast cancer is more common in young as compared to older women, an observation which is compatible with sexual transmission of HPV among sexually active younger women [36], (vi) HPV positive koilocytes have been identified in breast cancers [37], (vii) the prevalence of HPV positive breast cancer is higher in women with prior HPV associated cervical cancer [38], (viii) high risk HPV gene sequences are present in benign breast tissues prior to the development 3 to 10 years later of same HPV type positive breast cancers [4].

The prevalence of breast cancer is not increased in immunocompromised patients with AIDS or organ transplants [39]. This is in contrast to the 5 fold increase in HPV associated cervical cancer in these patients [39]. The implication is that any oncogenic influences of viruses in breast cancer are indirect or there is a “hit and run” viral influence. There is evidence in support of both notions. HPVs appear to influence the cell cycle control enzyme APOBEC which leads to genomic instability and ultimately to breast cancer [40]. There is high HPV E7 oncoprotein expression in benign breast tissues and low HPV E7 expression in subsequent breast cancer which developed in the same patients [41].

These data are compatible with an indirect role for high risk HPVs in breast cancer.

### Epstein Barr virus (EBV)

An association between EBV and breast cancer is likely. The evidence is as follows: (i) Various EBV genes have been identified by in situ hybridisation, immunohistochemistry, in situ PCR and by PCR in microdissected breast cancers in a wide range of countries [42]. The prevalence of EBVs in breast cancers varies from 0 to 100%. The identification of EBV in normal and benign breast tissue controls are, with 2 exceptions, negative [5, 42]. Many additional studies based on PCR of EBVs in breast cancer, are not valid because standard PCR techniques cannot distinguish between cancer cells and infiltrating lymphocytes, (ii) breast epithelial cells can be infected with EBV by cell to cell contact [43]. (iii) EBV infection predisposes breast epithelial cells to malignant transformation, [44], (iv) EBV is accepted as a major contributor to Hodgkin lymphoma and there are significant correlations between the incidence of Hodgkin lymphoma and breast cancer which suggests that EBV may also contribute to some breast cancers [45].

These observations are also compatible with an indirect role for EBV in breast cancer.

With respect to infectious agents and cancer, a key causal criteria is the presence of infectious agents prior to the development of cancer [46]. For this reason we have combined the data we developed during investigations of the individual four oncogenic viruses and breast cancer [2–5]. Here we show that multiple oncogenic viruses may be present in benign breast tissues prior to the development of the same virus positive invasive breast cancers in the same patients.

## Methods

This project has formal ethics approval by the University of New South Wales, Sydney, Australia, Human Research Ethics Committee – number HREC HC11421. De-identified archival specimens were used in this study.

Approximately 4000 patient records from a large Australian pathology service (Douglas Hanly Moir Pathology) were reviewed to identify patients who had breast biopsies that included an initial benign breast specimen and a later breast cancer specimen. Forty one suitable patients were identified. Archival formalin fixed specimens from these patients were collected. The average age of the patients with benign breast conditions was 50.5 years and subsequent cancer 56.1 years. The same archival specimens were used for (i) investigations of mouse mammary tumour virus (also known as human mammary tumour virus) conducted at the Icahn School of Medicine at Mount Sinai, New York and at the University of Pisa, Italy, (ii) bovine leukemia virus conducted at the University of California at Berkeley,(iii) human papilloma virus and Epstein Barr virus conducted at the University of New South Wales, Sydney, Australia.

Seventeen normal breast tissues from cosmetic breast surgery conducted on Australian patients were used as controls. These patients were younger than those with benign and later breast cancer.

Standard and/or in situ polymerase chain reaction (PCR) methods were used to identify the four viruses. The detailed methods are outlined in the separate publications: mouse mammary tumor virus, human papilloma virus and Epstein Barr virus [2–5]. A summary of the methods used is as follows:

### Detection of mouse mammary tumour virus-like env sequences

The DNA extraction and detection of MMTV-like *env* sequences were performed by PCR techniques as described by Wang et al. [47]. The primer sequences used in these PCR analyses include part of the MMTV *env* gene, which differs from human endogenous retrovirus 10 (HERV-K10). The same PCR techniques were used in both the Mount Sinai and University of Pisa laboratories with the exception of microdissection of the tumour tissues, that were analysed in the University of Pisa laboratory by fluorescence nested PCR.

### Detection of bovine leukemia virus sequences

Both standard and in situ PCR was used to detect BLV DNA in the tissue samples [3]. The primer sequences, were from the *tax* region of the BLV genome. The specificity of these primers for BLV has previously been demonstrated by NCBI BLAST sequence alignments [23].

### Detection of high risk for cancer human papilloma virus gene sequences

In situ PCR, semi-nested PCR, and real-time PCR plus whole genome sequencing were used for the detection of HPV [4]. All PCR products were sequenced to help identify any contamination. Although in situ PCR can produce false positive outcomes, use of this method can add to the validity of results based on semi-nested and real time PCR. The HPV PCR products from GP5 to Gp6 were sequenced to determine the HPV type. The HPV genotypes were identified by BLAST via the US National Center for Biotechnology Information.

### Detection of Epstein Barr gene sequences

Both standard and nested PCR and in situ PCR techniques were used [5].

## Results

The results are shown in Tables 1, 2 and 3. There was sufficient archival material to seek to identify the four target viruses in both benign breast tissues and subsequent breast cancers in the same patients in up to 27 of the 41 patients.

**Table 1:** Multiple viruses human breast cancers

Patient	Age	Diagnosis	BLV	MMTV	HPV	EBV
1	36	benign	pos	neg	neg	neg
	41	Invasive dc	pos	neg	18	neg
2	72	benign	pos	neg		
	75	dcis	pos	neg		
3	33	benign	pos	neg	18/45	
	44	Invasive dc	pos	neg	18	pos
4	45	benign	pos	pos	16/18	
	46	dcis	pos	pos	18	
5	50	benign	neg	pos	neg	
	60	Invasive lc	pos	pos	neg	neg
6	62	benign	pos	neg	124	neg
	66	dcis	pos	neg		neg
7	47	benign	pos	neg	neg	
	56	Invasive dc	pos	neg	neg	
8	49	benign	neg	neg		
	52	Invasive dc	pos	neg		
9	46	benign	pos	neg	18	
	53	Invasive dc	pos	pos	18	
10	48	hyperplasia	pos	pos	18	
	52	Invasive dc	pos	pos	neg	neg
11	35	benign	pos	neg		
	46	Invasive dc	neg	neg		
12	44	benign	pos	neg	18	neg
	48	Invasive dc	neg	neg	18	neg
13	67	hyperplasia	neg	neg		
	75	Invasive dc		neg		
14	48	benign		neg	neg	
	54	Invasive dc		neg	neg	pos
15	47	benign			18	
	48	Invasive dc			18	
16	42	benign	neg	neg	18	neg
	49	Invasive dc	pos	pos	18	neg
17	42	benign	pos	neg		
	48	Invasive dc	pos	neg		
18	39	benign	pos	neg	18	
	45	Invasive lc	pos	neg	18	
19	54	benign	pos	neg		
	62	dcis	pos	pos	18	
20	65	hyperplasia	pos	pos		neg
	67	Invasive dc	pos	neg		
21	39	benign	neg	neg	neg	
	44	dcis	pos	neg	neg	
22	55	benign	pos	neg		
	62	Invasive dc	pos	neg		
23	59	benign			18	
	65	Invasive lc			18	
24	37	benign	pos	neg	neg	
	39	Invasive dc	pos	neg	16/18	
25	39	benign		pos	18	
	42	dcis		pos	18	neg
26	48	benign	pos	pos	16/18	
	54	Invasive dc	pos	pos	16/18	pos
27	63	benign	pos	neg		
	67	Invasive dc	pos	pos	18	neg

*Dcis* ductal carcinoma in situ, *Invasive dc* invasive ductal carcinoma, *Invasive lc* invasive lobular carcinoma, *Pos* positive, *Neg* negative

Gaps in the table are due to inadequate tissues or absence of beta globin

**Table 2:** Multiple oncogenic viruses in benign breast and same virus in subsequent breast cancer in the same patients

	Benign breast biopsy tissues	Subsequent breast cancer same patient
Mouse mammary tumor virus	6/25 (24%)	9/25 (36%)
Bovine leukemia virus	18/23 (78%)	20/22 (91%)
High risk human papilloma virus	13/18 (72%)	13/17 (76%)
Epstein Barr virus	0/4 (0%)	3/12 (25%)

**Table 3:** MMTV, BLV, HPV and EBV sequences in normal breast tissues

Patient	Age	MMTV	BLV	HPV	EBV
1	22	neg	pos	neg	neg
2	24	neg	pos	neg	neg
3	26	neg	neg	neg	neg
4	46	pos	pos	pos	neg
5	49	neg	neg	neg	neg
6	26	neg	pos	neg	neg
7	46	pos	pos	neg	pos
8	27	neg	neg	neg	neg
9	55	neg	pos	neg	neg
10	61	neg	neg	neg	neg
11	28	pos	neg	pos	pos
12	28	neg	neg	neg	neg
13	19	neg	neg	neg	pos
14	55	neg	neg	neg	pos
15	33	neg	neg	pos	
16	19	neg	neg	neg	pos
17	55	neg	neg	neg	neg

Normal breast tissues were from cosmetic surgery

Bovine leukemia virus (BLV) was identified by in situ PCR in 18 (78%) of 23 benign breast specimens and in 20 (91%) of 22 subsequent breast cancers in the same patients. The identity of BLV was confirmed by sequencing the products of standard PCR. Because the prevalence of bovine leukemia virus is so high in both benign breast and later breast cancer cells (78% of benign breast and 91% of breast cancer specimens), it is likely that this virus is co-located with other virus positive benign and breast cancer cells.

Mouse mammary tumour virus was identified in 6 (24%) of 25 benign breast specimens and in 9 (36%) of 25 breast cancer specimens which subsequently developed in the same patients.

High risk human papilloma viruses were identified in 13 (72%) of 17 benign breast specimens and in 13 (76%) of 17 subsequent breast cancers in the same patients.

Epstein Barr virus was identified in 3 (25%) of 12 breast cancer specimens, but was not identified in any benign breast specimens from the same patients.

**Fig. 1:**
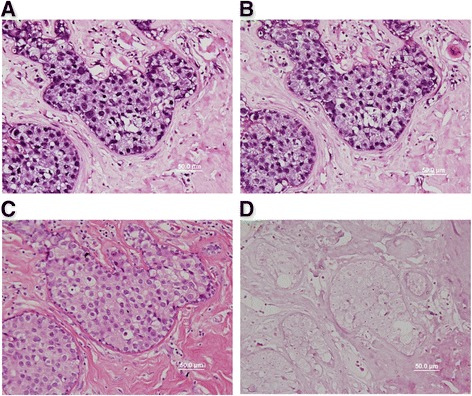
EBV and HPV nucleotide sequences shown by in situ PCR in the nuclei of the same breast cancer cells (ductal carcinoma in situ). **a** EBV in breast cancer cell nuclei. **b** HPV in breast cancer cell nuclei. **c** Haematoxylin and eosin (H & E) stain. **d** Negative controls. No PCR primers

EBV and HPV were identified in the same breast cancer cells by in situ PCR. The images are shown in Fig. 1.

MMTV 3 (18%), BLV 6 (35%), high risk HPV 3 (18%) and EBV 5 (29%) were identified in 17 normal control breast specimens.

These data indicate that multiple oncogenic viruses may be present in both prior benign breast tissues and subsequent breast cancers that developed 1 to 11 years later in the same patients. In addition, these same viruses are present in a small proportion of normal breast tissues.

The numbers of patients and specimens are too small to justify a formal assessment of correlations between the presence of one or more of these viruses.

## Discussion

In this study we have demonstrated the presence of four oncogenic viruses in benign breast tissues and the same viruses in subsequent breast cancers that developed 1 to 11 years later in the same patients. We have also shown that HPVs and EBV can be co-located in the same breast cancer cells.

Validity of the data in the current studies. PCR methods were used to identify the four oncogenic viruses, bovine leukemia virus, mouse mammary tumor virus, high risk human papilloma viruses and Epstein Barr virus. PCR analyses are notoriously liable to contamination leading to false positive outcomes. Not so well known is the problem of false negative outcomes when PCR methods are used to identify low concentrations of retroviruses. This latter issue has been considered in detail by Vinner et al. [26]. Several different approaches were made to overcome these problems. With respect to bovine leukemia virus in situ PCR methods were used. As shown in Fig. 1, this method has the advantage of distinguishing between breast cancer and other cells. The identity of BLV was confirmed by the sequencing of the PCR products and comparisons with standard reference sequences. With respect to mouse mammary tumour virus, standard PCR methods were used on the same specimens in two independent laboratories. In the Pisa laboratories laser microdissection techniques were used to carefully exclude stromal and lymphocyte cells from the breast tissues. In the Mount Sinai, New York, laboratories PCR methods were used to exclude contamination by mouse mitochondrial sequences. However it should be noted that the results of PCR analyses of the same breast specimens has some differences between the two laboratories. This is a reflection of the difficulty of consistent detection of low concentrations of retroviruses. With respect to high risk human papilloma viruses, the identification was by in situ PCR, standard PCR and immunohistochemistry, each of which added to the validity of the outcomes. There were variations in the sequences which is an indication that contamination was unlikely. With respect to Epstein Barr virus the identification was also by in situ PCR, standard PCR and immunohistochemistry, each of which added to the validity of the outcomes.

There is substantial, but not conclusive, evidence that each of these viruses may have oncogenic influences in breast cancer. This is despite the fact that in immunocompromised patients such as those with organ transplant immunosuppression or human immunodeficiency virus, there is no increased prevalence of virus positive breast cancer [39]. It has been argued that, if for example MMTVs or HPVs had a causal role in breast cancer, an increase in expected prevalence should occur as there is an up to 5 fold increase in HPV associated cervical cancer among these patients. The explanation with respect to MMTV is that intact lymphocytes are required to transmit the virus to target organs [7]. Lymphocytes, as part of the immune system, are compromised in immunosuppressed patients. With respect to HPVs, their oncogenic influences may be indirect and not subject to the immune system. HPVs increase the activity of the cell cycle enzyme APOBEC3B which in turn causes genomic instability and increased risk of breast and other cancers [40].

There are a number of different types of breast cancer. Historically these types have been classified mainly by their histological characteristics and include ductal carcinoma in situ, invasive ductal carcinoma, invasive lobular carcinoma and special types including tubular carcinoma, cribriform carcinoma, mucinous carcinoma, medullary carcinoma, micropapillary carcinoma, neuro-endocrine carcinoma plus other rare types [48]. More recently invasive breast cancers have been classified by their molecular characteristics and include luminal, basal like and HER 2 status [49]. It is possible that different viruses are associated with different types of breast cancer. For example mouse mammary tumour virus positive invasive human breast cancers frequently have the same histological characteristics as mouse mammary virus positive mouse mammary tumours. These tumours are very similar to many breast ductal carcinoma in situ breast cancers and special breast cancer types such as medullary carcinoma, adenoid cystic carcinoma and neuro-endocrine carcinoma [50, 51].

Several of these four oncoviruses are co-located in breast cancer cells and may collaborate with each other to increase their oncogenic potential. We have shown in this current study that HPVs and EBV can be co-located in breast cancer cells. We have previously shown that MMTV gene sequences may also be co-located with HPVs and EBVs in the same breast cancer cells [5]. Because the prevalence of bovine leukemia virus is high in both benign breast and later breast cancer cells (78% of benign breast and 91% of breast cancer specimens), it is likely that this virus is also co-located with other virus positive benign and breast cancer cells. However there is no direct evidence of such co-location. Nor is there evidence that such co-location of these viruses leads to increased oncogenic influences in breast cancer. On the other hand, in studies of women with breast cancer in Syria, it was shown that a co-prevalence of EBVs and high risk HPVs in 32% of the breast tumours was associated with high grade invasive ductal breast cancers [52]. In addition there is preliminary experimental evidence that HPVs and EBVs collaborate [53]. It is not known how different viruses co-operate to induce cancer. There are current research activities aimed at the elucidation of this issue with specific reference to HPV and EBV [54].

## Conclusions

The identification of multiple oncogenic viruses in benign breast tissues prior to the subsequent development of same virus breast cancer in the same patients is an important observation. This is because evidence of prior infection before the development of disease is a key criterion when assessing causation. These findings add to the evidence that oncogenic viruses have potential roles in human breast cancer.
